# Overall survival is improved when DCIS accompanies invasive breast cancer

**DOI:** 10.1038/s41598-019-46309-2

**Published:** 2019-07-09

**Authors:** Adam J. Kole, Henry S. Park, Skyler B. Johnson, Jacqueline R. Kelly, Meena S. Moran, Abhijit A. Patel

**Affiliations:** 1 Department of Therapeutic Radiology, Yale University School of Medicine, New Haven, CT USA; 2 Present Address: Department of Radiation Oncology, University of Alabama at Birmingham, Birmingham, AL USA

**Keywords:** Oncogenesis, Breast cancer, Breast cancer

## Abstract

Invasive ductal carcinoma (IDC) often presents alone or with a co-existing ductal carcinoma *in situ* component (IDC + DCIS). Studies have suggested that pure IDC may exhibit different biological behavior than IDC + DCIS, but whether this translates to a difference in outcomes is unclear. Here, utilizing the National Cancer Database we identified 494,801 stage I-III breast cancer patients diagnosed with either IDC alone or IDC + DCIS. We found that IDC + DCIS was associated with significantly better overall survival (OS) compared to IDC alone (5-year OS, 89.3% vs. 85.5%, p < 0.001), and this finding persisted on multivariable Cox modeling adjusting for demographic, clinical, and treatment-related variables. The significantly superior OS observed for IDC + DCIS was limited to patients with invasive tumor size < 4 cm or with node negative disease. A greater improvement in OS was observed for tumors containing ≥25% DCIS component. We also found IDC + DCIS to be associated with lower T/N stage, low/intermediate grade, ER/PR positivity, and receipt of mastectomy. Thus, the presence of a DCIS component in patients with IDC is associated with favorable clinical characteristics and independently predicts improved OS. IDC + DCIS could be a useful prognostic factor for patients with breast cancer, particularly if treatment de-escalation is being considered for small or node negative tumors.

## Introduction

Ductal carcinoma *in situ* (DCIS) is an established precursor to invasive breast cancer and often co-exists pathologically with invasive ductal carcinoma (IDC)^1–3^. Currently, treatment paradigms for such cases of IDC with a DCIS component (IDC + DCIS) are similar to those for pure IDC alone, with the extent and characteristics of invasive disease driving clinical decisions^4^. It remains unclear, however, whether survival outcomes are similar for IDC when it presents alone or is accompanied by co-existing DCIS.

Studies have explored whether IDC + DCIS may be biologically distinct from IDC alone^5–9^. It has been hypothesized that tumors present as combined IDC + DCIS when the progression from pre-invasive DCIS to IDC is delayed – a sign of reduced biological aggressiveness^6^. In contrast, tumors presenting as IDC alone may have achieved invasive potential early in the process of carcinogenesis, leaving little or no evidence of the pre-invasive state. Indeed, prior studies have demonstrated that IDC + DCIS tumors are associated with favorable clinical characteristics such as smaller tumor size, lower tumor grade, lower Ki-67 staining, greater ER-positivity, and reduced risk of local recurrence when compared to IDC alone^5–9^. While trends towards improved overall survival (OS) have been previously observed with IDC + DCIS versus IDC alone^5,6,9^, the limited sample sizes of prior studies likely did not provide sufficient power to detect a statistically significant OS difference.

Here, we took advantage of the large data set available in the National Cancer Database (NCDB) to assess whether significant differences in OS exist between patients with IDC alone versus those with IDC + DCIS. The large sample size also enabled us to investigate whether the effect of DCIS on survival differed when patients were categorized by the size of their invasive tumor component, nodal stage, or extent of the DCIS component.

## Methods

### Data source

Patient data were obtained from the NCDB, which is a nationwide, hospital-based patient registry established as a joint project of the American College of Surgeons Commission on Cancer and the American Cancer Society. The NCDB captures approximately 70% of all new cancer diagnoses in the United States. A de-identified NCDB file was used to obtain data for this study. This work has not been verified by the American College of Surgeons or the American Cancer Society and these societies are not responsible for the methodology or conclusions drawn from this work.

### Study population

**Figure 1:**
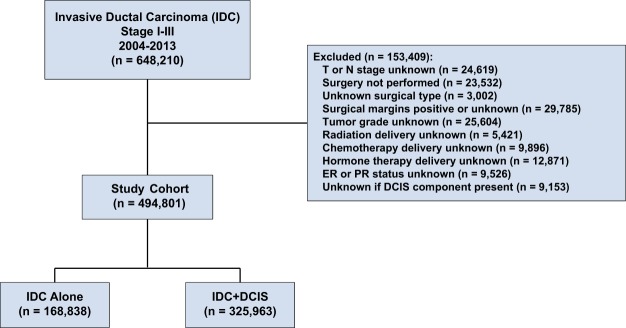
Study cohort flow diagram. Abbreviations: DCIS, ductal carcinoma *in situ*; IDC, invasive ductal carcinoma; IDC + DCIS, invasive ductal carcinoma with ductal carcinoma *in situ*.

Clinical stage I-III breast cancer patients diagnosed from 2004 to 2013 were identified in the NCDB. Patients were required to have histologically-confirmed invasive ductal carcinoma and no prior cancer diagnosis. A total of 648,210 patients met these initial criteria. Figure 1 shows the inclusion and exclusion criteria that resulted in our main study cohort of 494,801 patients. All patients underwent lumpectomy or mastectomy with or without axillary lymph node dissection.

Patients were excluded if any of the following parameters were coded as unknown: clinical T or N stage, pathologic surgical margin status, tumor grade, estrogen receptor (ER) and progesterone receptor (PR) status, receipt of any treatment variables (radiation, chemotherapy, or hormone therapy), or if it was unknown whether a DCIS component was present (using the CS Site-Specific Factor 6 variable within the NCDB participant user file). Patients with positive margins were excluded, as the NCDB dataset did not specify whether this referred to positive invasive or *in situ* margins. Her2 was not consistently reported within the NCDB prior to 2010, thus unknown Her2 status was not used as an exclusion criterion.

### Statistical analysis

Univariable logistic regression was used to calculate unadjusted odds ratios (ORs) for the diagnosis of IDC alone versus IDC + DCIS based on categorized baseline clinical and pathologic factors. Factors included patient age (<60 vs. ≥60 years), race/ethnicity (white vs. non-white); Charlson-Deyo co-morbidity score (0 vs. ≥1); clinical T stage (T1 vs. T2, T3, or T4) and N stage (N0 vs. N1, N2, or N3); ER (positive vs. negative), PR (positive vs. negative), and Her2 (negative vs. positive or unknown) receptor status; surgical type (partial mastectomy vs. mastectomy); and tumor grade (low/intermediate vs. high). As the extent of surgery could affect the available tissue for pathological examination, surgical type was included in the Cox regression. Other treatment variables (radiation therapy, chemotherapy, hormone therapy), were excluded from OR calculations as these treatments are typically prescribed after the diagnosis of IDC alone or IDC + DCIS would have been made. Factors with a trend towards significance (p < 0.10) were included in a multivariable regression analysis. A factor was considered to be a statistically significant predictor of diagnosis of IDC alone versus IDC + DCIS if p < 0.05. Patients with missing data (eg. Her2 status) were included in univariable and multivariable analyses using an “unknown” dummy variable. A sensitivity analysis was performed in which patient age was either used as a continuous variable or dichotomized at age 40, 50, 70, or 80 to evaluate the impact on results of the multivariable Cox regression.

OS was compared between IDC alone and IDC + DCIS groups using the Kaplan-Meier method and log-rank test. Univariable and multivariable Cox regressions were used to calculate unadjusted and adjusted hazard ratios (HRs) for survival, respectively. As described above, all clinical and pathologic variables with p < 0.10 on univariable analysis were included in the multivariable model. The proportional hazards assumption was checked graphically using log-log survival plots.

To further examine survival differences between patients with IDC alone versus IDC + DCIS, four additional analyses were performed. The first was conducted on patients with known size of the invasive tumor component, excluding any tumors ≥7 cm as it was felt these large tumor sizes represented coding errors. Patients were categorized to the following invasive tumor size groups: <1 cm, 1 to <2 cm, 2 to <3 cm, 3 to <4 cm, 4 to <5 cm, or 5 to <7 cm. A second analysis was performed by categorizing patients based on clinical node status: N0, N1, N2, or N3. A third analysis evaluated patients when grouped into the following 3 biologic subtypes: (1) ER or PR positive, Her2 negative; (2) triple negative; (3) Her2 positive. Finally, a fourth analysis was performed among patients for which the extent of DCIS was known to be “minimal” or “extensive” (defined as a DCIS component of <25% or ≥25% of the total tumor size, respectively). While an extensive intraductal component (EIC) has been associated with higher rates of local recurrence when margins are positive^10–13^, our study cohort was limited to patients with negative margins. Of note, the CS Site-Specific Factor 6 variable in the NCDB either codes patients by known invasive tumor component size or by extent of DCIS, but not both. Thus, the IDC + DCIS patients analyzed by invasive tumor size or by extent of DCIS represented independent cohorts. For the first three subgroup analyses, independent multivariable Cox regressions were performed to calculate adjusted HRs (aHRs) for IDC + DCIS versus IDC alone for each group. The Kaplan-Meier method and log-rank test were used for the fourth subgroup to compare OS between patients with a minimal DCIS component, extensive DCIS, or IDC alone.

STATA/SE version 13.1 (Stata Corp, College Station, TX) was used for all statistical analyses. All tests were two-sided, and a p value of <0.05 was considered statistically significant.

### Ethics approval and informed consent

The National Cancer Database provides a Participant Use Data File (PUF) which is compliant with the Health Insurance Portability and Accountability Act (HIPAA). Data in the PUF are de-identified with regards to the patient and treatment facility. Institutional Review Board (IRB) approval was not required.

## Results

### Patient and tumor characteristics

We identified a total of 494,801 stage I-III breast cancer patients with either IDC alone or IDC + DCIS who met study inclusion criteria (Fig. 1). Patient demographic and clinical-pathologic characteristics are shown in Table 1. Median follow-up was 4.5 years. Fewer patients were diagnosed with IDC alone (168,838 patients; 34%) than with IDC + DCIS (325,963 patients; 66%). The median age for the study cohort was 59.7 years. The majority of patients were white (74.9%), with T1 (65.8%) and N0 (82.7%) tumors, with receptor status ER positive (78.3%), and PR positive (68.9%). Among patients with known Her2 status, the majority had Her2 negative disease (83.3%). Partial mastectomy was performed in 61.6% of patients while 38.4% received mastectomy. The majority of patients received radiation therapy (65.9%) and hormonal therapy (73.7%). Approximately one half of patients (48.9%) received chemotherapy. The receipt of radiation and systemic therapies in the setting of partial or total mastectomy are presented for node-positive and node-negative patients in Supplementary Table 1.

**Table 1:** Demographic and clinical-pathologic characteristics of patient cohort.

Characteristic	IDC alone (n = 168,838) No. (%)	IDC + DCIS (n = 325,963) No. (%)	Total (n = 494,801) No. (%)
**Age (years):**			
Mean:	60.4	59.3	59.7
**Categorized:**			
<40	8,941 (5.3)	16,961 (5.2)	25,902 (5.2)
40 to 49	28,913 (17.1)	65,196 (20.0)	94,109 (19.0)
50 to 59	43,149 (25.6)	86,662 (26.6)	129,811 (26.2)
60 to 69	44,176 (26.2)	83,349 (25.6)	127,525 (25.8)
70 to 79	28,342 (16.8)	50,181 (15.4)	78,523 (15.9)
≥80	15,317 (9.1)	23,614 (7.2)	38,931 (7.9)
**Race/Ethnicity:**			
White	125,149 (74.1)	245,641 (75.4)	370,790 (74.9)
Black	19,898 (11.8)	31,542 (9.7)	51,440 (10.4)
Hispanic	7,808 (4.6)	14,652 (4.5)	22,460 (4.5)
Other	15,524 (9.2)	33,259 (10.2)	48,783 (9.9)
Unknown	459 (0.3)	869 (0.3)	1,328 (0.3)
**Charlson-Deyo Co-morbidity Score:**			
0	142,640 (84.5)	276,583 (84.9)	419,223 (84.7)
1	21,527 (12.8)	40,876 (12.5)	62,403 (12.6)
2	4,671 (2.8)	8,504 (2.6)	13,175 (2.7)
**T Stage:**			
T1	101,048 (59.9)	224,503 (68.9)	325,551 (65.8)
T2	52,136 (30.9)	85,218 (26.1)	137,354 (27.8)
T3	9,400 (5.6)	11,379 (3.5)	20,779 (4.2)
T4	6,254 (3.7)	4,863 (1.5)	11,117 (2.3)
**N Stage:**			
N0	133,919 (79.3)	275,267 (84.5)	409,186 (82.7)
N1	26,357 (15.6)	40,668 (12.5)	67,025 (13.6)
N2	5,979 (3.5)	7,145 (2.2)	13,124 (2.7)
N3	2,583 (1.5)	2,883 (0.9)	5,466 (1.1)
**ER Status:**			
Positive	120,952 (71.6)	266,589 (81.8)	387,541 (78.3)
Negative	47,886 (28.4)	59,374 (18.2)	107,260 (21.7)
**PR Status:**			
Positive	105,085 (62.2)	235,770 (72.3)	340,855 (68.9)
Negative	63,753 (37.8)	90,193 (27.7)	153,946 (31.1)
**Her2 Status:**			
Positive	13,448 (8.0)	33,962 (10.4)	47,410 (9.6)
Negative	79,866 (47.3)	156,994 (48.2)	236,860 (47.9)
Unknown	75,524 (44.7)	135,007 (41.4)	210,531 (42.6)
**Surgery Type:**			
Partial Mastectomy	107,159 (63.5)	197,648 (60.6)	304,807 (61.6)
Mastectomy	61,679 (36.5)	128,315 (39.4)	189,994 (38.4)
**Radiation Therapy:**			
No	54,619 (32.4)	114,151 (35.0)	168,770 (34.0)
Yes	114,219 (67.7)	211,812 (65.0)	326,031 (65.9)
**Chemotherapy:**			
No	81,704 (48.4)	171,087 (52.5)	252,791 (51.1)
Yes	87,134 (51.6)	154,876 (47.5)	242,010 (48.9)
**Hormone Therapy:**			
No	55,069 (32.6)	75,007 (23.0)	130,076 (26.3)
Yes	113,769 (67.4)	250,956 (77.0)	364,725 (73.7)
**Tumor Grade:**			
Low	35,388 (21.0)	67,934 (20.8)	103,322 (20.9)
Intermediate	61,456 (36.4)	143,865 (44.1)	205,321 (41.5)
High	71,994 (42.6)	114,164 (35.0)	186,158 (37.6)

Abbreviations: IDC, invasive ductal carcinoma; IDC + DCIS, invasive ductal carcinoma with ductal carcinoma *in situ*.

Percentages may not add to 100.0% due to rounding.

### Factors associated with the presence of a dcis component

Factors which were statistically associated with IDC + DCIS diagnosis versus IDC alone on multivariable analysis included: age <60 years, lower T and N stage, ER positivity, PR positivity, and lower tumor grade (Table 2). Patients who underwent mastectomy versus partial mastectomy were more likely to have a diagnosis of IDC + DCIS. Patient race/ethnicity and Charlson-Deyo co-morbidity score were not significant on multivariable analysis (Table 2).

**Table 2:** Univariable and multivariable logistic regression to determine odds of diagnosis with IDC + DCIS versus IDC alone.

Variable	Univariable Analysis			Multivariable Analysis		
	OR	95% CI	p value	aOR	95% CI	p value
**Age (Ref: <60):**						
≥60	0.86	0.85–0.87	<0.001	0.80	0.79–0.81	<0.001
**Race (Ref: White):**						
Non-White	0.94	0.92–0.95	<0.001	1.01	0.99–1.02	0.199
**Charlson-Deyo Score (Ref: 0):**						
≥1	0.97	0.96–0.99	0.001	1.00	0.99–1.02	0.624
**T Stage (Ref: T1):**						
T2	0.74	0.73–0.75	<0.001	0.75	0.74–0.76	<0.001
T3	0.54	0.53–0.56	<0.001	0.55	0.53–0.57	<0.001
T4	0.35	0.34–0.36	<0.001	0.36	0.35–0.38	<0.001
**N Stage (Ref: N0):**						
N1	0.75	0.74–0.76	<0.001	0.86	0.85–0.88	<0.001
N2	0.58	0.56–0.60	<0.001	0.74	0.71–0.77	<0.001
N3	0.54	0.51–0.57	<0.001	0.73	0.69–0.78	<0.001
**ER Status (Ref: Positive):**						
Negative	0.56	0.55–0.57	<0.001	0.68	0.67–0.70	<0.001
**PR Status(Ref: Positive):**						
Negative	0.63	0.62–0.64	<0.001	0.85	0.84–0.87	<0.001
**Her2 Status (Ref: Negative):**						
Positive	1.28	1.26–1.31	<0.001	1.48	1.45–1.51	<0.001
Unknown	0.91	0.90–0.92	<0.001	0.95	0.94–0.97	<0.001
**Surgery Type (Ref: Partial Mastectomy):**						
Mastectomy	1.13	1.11–1.14	<0.001	1.37	1.36–1.40	<0.001
**Tumor Grade (Ref: Low/Intermediate):**						
High	0.73	0.72–0.73	<0.001	0.93	0.92–0.95	<0.001

Abbreviations: aOR, adjusted odds ratio; CI, confidence interval; IDC, invasive ductal carcinoma; IDC + DCIS, invasive ductal carcinoma with ductal carcinoma *in situ*.

### Overall Survival for patients with IDC alone versus IDC + DCIS

**Figure 2:**
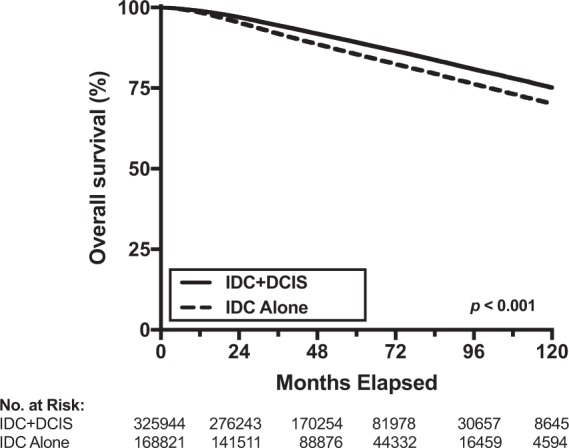
Overall survival. Kaplan-Meier curves comparing overall survival between patients with invasive ductal carcinoma (IDC) alone or IDC with a ductal carcinoma *in situ* component (IDC + DCIS).

In the primary cohort, the presence of IDC + DCIS was associated with significantly improved OS compared to IDC alone on univariable analysis (5-year OS, 89.3% vs. 85.5, p < 0.001; hazard ratio [HR], 0.74; 95% CI, 0.73–0.75, p < 0.001) (Fig. 2). A total of 14 variables were included in our multivariable Cox survival model: tumor histology (IDC vs. IDC + DCIS), age, race/ethnicity, Charlson-Deyo score, clinical T and N stage, ER status, PR status, Her2 status, surgery type, radiation therapy receipt, chemotherapy receipt, hormone therapy receipt, and tumor grade. When adjusting for these factors, IDC + DCIS remained associated with improved OS (HR 0.91, 95% CI 0.89–0.92, p < 0.001) (Table 3). Sensitivity analysis showed IDC + DCIS to remain significantly associated with improved OS when age was analyzed as a continuous variable or dichotomized at cut-points of 40, 50, 60, 70, or 80 years (data not shown).

**Table 3:** Univariable and multivariable Cox proportional hazards models to determine predictors of OS.

Variable	Univariable Analysis			Multivariable Analysis		
	HR	95% CI	p value	aHR	95% CI	p value
**Histology (Ref: IDC alone):**						
IDC + DCIS	0.74	0.73–0.75	<0.001	0.91	0.89–0.92	<0.001
**Age (Ref: <60):**						
≥60	2.29	2.25–2.33	<0.001	2.14	2.10–2.18	<0.001
**Race (Ref: White):**						
Non-White	1.07	1.05–1.09	<0.001	0.97	0.95–0.98	<0.001
**Charlson-Deyo Score (Ref: 0):**						
≥1	2.15	2.11–2.19	<0.001	1.71	1.68–1.74	<0.001
**T Stage (Ref: T1):**						
T2	2.00	1.96–2.03	<0.001	1.86	1.83–1.90	<0.001
T3	3.10	3.01–3.20	<0.001	2.93	2.83–3.03	<0.001
T4	5.17	5.00–5.34	<0.001	3.92	3.77–4.07	<0.001
**N Stage (Ref: N0):**						
N1	1.84	1.81–1.88	<0.001	1.67	1.63–1.71	<0.001
N2	2.82	2.73–2.92	<0.001	2.29	2.20–2.37	<0.001
N3	4.06	3.87–4.25	<0.001	3.10	2.95–3.26	<0.001
**ER Status (Ref: Positive):**						
Negative	1.89	1.86–1.92	<0.001	1.11	1.07–1.14	<0.001
**PR Status(Ref: Positive):**						
Negative	1.82	1.79–1.85	<0.001	1.26	1.22–1.29	<0.001
**Her2 Status (Ref: Negative):**						
Positive	0.96	0.92–1.00	0.053	0.82	0.79–0.85	<0.001
Unknown	1.09	1.07–1.10	<0.001	1.00	0.98- 1.02	0.846
**Surgery Type (Ref: Partial Mastectomy):**						
Mastectomy	1.75	1.73–1.78	<0.001	0.92	0.90–0.94	<0.001
**Radiation Therapy (Ref: No):**						
Yes	0.52	0.51–0.53	<0.001	0.57	0.56–0.59	<0.001
**Chemotherapy (Ref: No):**						
Yes	0.84	0.83–0.86	<0.001	0.53	0.52–0.54	<0.001
**Hormone Therapy (Ref: No):**						
Yes	0.51	0.50–0.52	<0.001	0.73	0.71–0.75	<0.001
**Tumor Grade (Ref: Intermediate):**						
Low	0.75	0.73–0.77	<0.001	0.81	0.79–0.84	<0.001
High	1.64	1.61–1.67	<0.001	1.36	1.34–1.39	<0.001

Abbreviations: aHR, adjusted hazard ratio; ALND, axillary lymph node dissection; CI, confidence interval; HR, hazard ratio; IDC, invasive ductal carcinoma; IDC + DCIS, invasive ductal carcinoma with ductal carcinoma *in situ*; OS, overall survival.

### Survival outcomes based on invasive tumor size and nodal status

**Figure 3:**
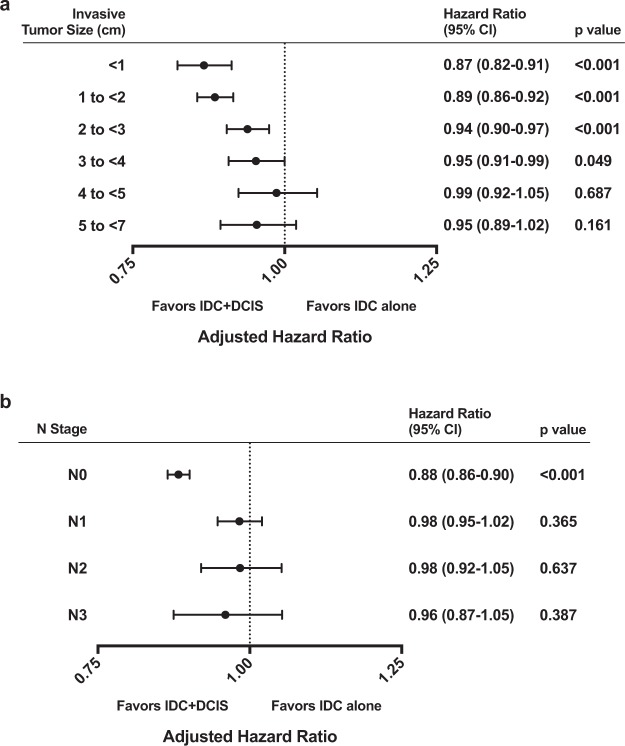
Survival trends based on tumor size and nodal status. Adjusted hazard ratios illustrating the effect of IDC alone vs. IDC + DCIS on overall survival when patients were categorized by (**a**) size of invasive component and (**b**) nodal stage. Abbreviations: CI, confidence interval; DCIS, ductal carcinoma *in situ*; IDC, invasive ductal carcinoma; IDC + DCIS, invasive ductal carcinoma with ductal carcinoma *in situ*.

For patients in whom the invasive tumor size was known (85.3% of the cohort; 422,227 patients) we repeated the analysis using our multivariable model after categorizing patients into one of six invasive tumor size groups: <1 cm, 1 to <2 cm, 2 to <3 cm, 3 to <4 cm, 4 to <5 cm, or 5 to <7 cm (Supplementary Table 2). As shown in Fig. 3a, OS was better for IDC + DCIS compared to IDC alone for the categories of patients with invasive tumor size less than 4 cm (<1 cm: HR, 0.87; 95% CI, 0.82–0.91; p < 0.001; 1 to <2 cm: HR, 0.89; 95% CI, 0.86–0.92; p < 0.001; 2 to <3 cm: HR, 0.94; 95% CI, 0.90–0.97; p < 0.001; 3 to <4 cm: HR, 0.95; 95% CI, 0.91–0.99; p = 0.049). For invasive tumors categorized as 4 cm or larger, survival outcomes between patients with IDC + DCIS and IDC alone were not statistically different.

The association of OS with IDC + DCIS versus IDC alone was also tested in our multivariable survival model when categorizing patients based on clinical node status (N0, N1, N2, or N3). Figure 3b shows that OS was improved with IDC + DCIS for patients with N0 disease (HR, 0.88; 95% CI, 0.86–0.90; p < 0.001), but not for patients with node positive (N1–N3) disease.

### Association of survival with extent of DCIS and biologic subtype

**Figure 4:**
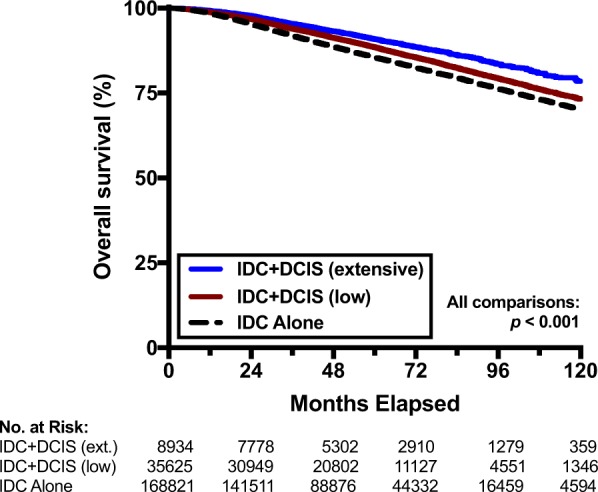
Overall survival according to extent of DCIS. Kaplan-Meier curves comparing overall survival among patients with invasive ductal carcinoma (IDC) alone, IDC with a low (<25%) DCIS component, or IDC with an extensive (≥25%) DCIS component.

Finally, we examined whether the extent of the DCIS component or biologic subtype influenced survival outcomes. Patients with IDC + DCIS with either a low (<25%) or extensive (≥25%) DCIS component were compared to patients with IDC alone. As local recurrence rates have been observed to be higher with an extensive intraductal component (EIC) unless appropriate resection with negative margins is achieved^10,12,13^, it is important to note that all patients had negative margins. Characteristics of the extent of DCIS are presented in Supplementary Table 3. Indeed, OS was better for patients who had a higher proportion of DCIS (Fig. 4), with 5-year OS rates of 85.5%, 88.5%, and 90.0% for IDC alone, low DCIS, and extensive DCIS, respectively (p < 0.001 for all comparisons). Supplementary Table 4 shows the biologic subtype among those patients for which ER, PR, and Her2 status are known. Interestingly, while IDC + DCIS was associated with improved survival for patients who were (1) ER or PR positive and Her2 negative or (2) Her2 positive, this was not seen for patients with triple negative disease (Supplementary Fig. 1).

## Discussion

In this study we show that for patients with IDC, the presence of an accompanying DCIS component is associated with favorable prognostic features and confers a statistically significant improvement in OS. Our finding that IDC + DCIS is associated with lower clinical stage, lower tumor grade, and greater ER and PR positivity is consistent with prior studies^5–8^. Despite these observed differences in tumor characteristics, however, prior studies failed to demonstrate a significant improvement in OS for patients with IDC + DCIS versus IDC alone, likely due to limited statistical power. The large size of our study, with nearly 500,000 patients, allowed us to identify a significant OS difference, and to also examine whether factors such as invasive tumor size and nodal status influence survival outcomes. We find that the association of IDC + DCIS with improved OS dissipates upon reaching 4 cm of invasive disease or node positivity, suggesting that at a given threshold of disease burden, a DCIS component is no longer predictive of improved survival.

The presence of an extensive intraductal component has traditionally been viewed as a negative prognostic factor for local recurrence in the setting of breast conservation^14–16^, likely due to the burden of residual DCIS in the breast^11^. In the setting of appropriate surgery with negative margins, however, the local recurrence risk with EIC is similar to that of non-EIC patients^10,12,13^. In our analysis of patients with negative margins, we find that OS rates are actually better when an extensive intraductal component (≥25% DCIS) is present compared to patients with a low (<25%) intraductal component. Patients with either extensive or low intraductal components had higher OS than those with IDC alone. These observations suggest that tumors with larger proportions of DCIS may indeed be less biologically aggressive.

Existing evidence suggests that biological differences may exist between breast cancers which present as IDC + DCIS versus IDC alone^6^. Extensive analyses of breast cancer genomics by The Cancer Genome Atlas (TCGA) Network and others have identified distinct molecular subtypes of breast cancer, but these have focused on IDC and have not explicitly evaluated situations when IDC is accompanied by DCIS^17–19^. The presence of appreciable *in situ* disease may indicate that a tumor underwent many cell divisions before a subclone evolved to acquire an invasive phenotype. One might speculate that slower evolution to invasiveness could be a mark of reduced genomic instability, which is known to portend a more favorable prognosis^20–22^. While our data are consistent with the hypothesis that IDC + DCIS may be inherently less biologically aggressive, the molecular underpinnings for these findings remain unknown. In fact, while our findings appear most applicable to patients with hormone receptor or Her2 positive disease, triple negative tumors may exhibit different behavior.

Can our findings prove useful in clinical practice? There is a growing interest to identify patient populations for which breast cancer treatment can be de-intensified. Among patients with DCIS alone, recent studies have identified patients at highest risk for progression to invasive disease^23–25^. For example, after publication of CALGB 9343, the National Comprehensive Cancer Network (NCCN) changed guidelines to support the omission of adjuvant RT in elderly patients with favorable disease^26,27^. Modern studies, such as the IDEA Study (Individualized Decisions for Endocrine Therapy Alone) and TAILORx (Trial Assigning Individualized Options for Treatment), have explored whether patients may omit adjuvant RT or adjuvant chemotherapy, respectively, in the context of favorable gene-expression molecular profiles^28,29^. Although the survival difference we observe between IDC and IDC + DCIS is small, the presence of accompanying DCIS could be considered for incorporation as an additional factor in predictive models to further refine the selection of patients eligible for treatment de-escalation.

Several limitations of our study should be noted. First, while the NCDB provides data regarding overall survival, cancer-specific outcomes such as local control, metastasis-free survival, and breast cancer-specific survival are unavailable. Second, while we quantitatively examined the effect of invasive tumor size on OS, the NCDB only records DCIS extent as a dichotomized variable (<25% or ≥25%). Thus, the minimum amount of DCIS that is necessary to observe an association with improved OS remains unclear. Third, because central pathologic review is not a requirement for NCDB data inclusion, inconsistencies in pathologic assessment across participating institutions could not be accounted for in this analysis. Fourth, biases inherent to the retrospective nature of our study may have been introduced. For example, selection bias is a frequent limitation in NCDB studies and could effect survival outcomes between study groups. However, it is likely that our study is less subject to selection bias as two groups are compared only based on a pathologic variable (presence or absence of a DCIS component) that should not significantly impact treatment decisions. Finally, the NCDB is not a population-based database^30^, thus patients presenting to NCDB-participating hospitals may not accurately represent the greater US population.

## Conclusion

We find that breast cancer survival is improved when DCIS accompanies IDC, particularly for patients with invasive disease measuring less than 4 cm or node-negative disease. These findings suggest that the presence of DCIS with IDC may be a marker of reduced aggressiveness, and could be incorporated as a prognostic feature in future treatment algorithms.

## Supplementary information

Supplementary Information
