# Supplementary material for: Overall survival is improved when DCIS accompanies invasive breast cancer
Source: Sci Rep. 2019 Jul 9;9:9934. doi: 10.1038/s41598-019-46309-2 (PMC6616329; doi:10.1038/s41598-019-46309-2)

Supplementary Information

Manuscript Title: Overall survival is improved when DCIS accompanies invasive breast cancer

Authors: Adam J. Kole, MD, PhD; Henry S. Park, MD, MPH; Skyler B. Johnson, MD; Jacqueline R. Kelly, MD, MSc; Meena S. Moran, MD; Abhijit A. Patel, MD, PhD

Contents:

1. Supplementary Table 1: Receipt of radiation and systemic therapies based on surgical type and nodal status.
2. Supplementary Table 2: Invasive tumor sizes among patients evaluated in matched invasive tumor size analysis
3. Supplementary Table 3: Extent of DCIS among patients with negative margins
4. Supplementary Table 4: Biologic subtypes among patients evaluated in subset analysis
5. Supplementary Figure 1: Adjusted hazard ratios illustrating the effect of IDC alone vs. IDC+DCIS on overall survival when patients were categorized by biologic subtype.

Abbreviations: CI, confidence interval; DCIS, ductal carcinoma in situ; IDC, invasive ductal carcinoma; IDC+DCIS, invasive ductal carcinoma with ductal carcinoma in situ.

Supplementary Table 1: Receipt of radiation and systemic therapies based on surgical type and nodal status.

Treatment received	IDC alone (n = 167,436) No. (%)	IDC+DCIS (n = 271,194) No. (%)
**Node-negative patients**:		
Partial mastectomy:		
Radiation		
No	13,189 (14.0)	21,070 (11.7)
Yes	81,032 (86.0)	159,061 (88.3)
Chemotherapy		
No	59,313 (63.0)	116,911 (64.9)
Yes	34,908 (37.1)	63,220 (35.1)
Hormonal therapy		
No	25,696 (27.3)	36,039 (20.0)
Yes	68,525 (72.7)	144,092 (80.0)
Mastectomy		
Radiation		
No	32,216 (81.2)	78,930 (83.0)
Yes	7,482 (18.9)	16,206 (17.0)
Chemotherapy		
No	17,756 (44.7)	46,455 (48.8)
Yes	21,942 (55.3)	48,681 (51.2)
Hormonal therapy		
No	14,590 (36.8)	24,563 (25.8)
Yes	25,108 (63.3)	70,573 (74.2)
**Node-positive patients**:		
Partial mastectomy:		
Radiation		
No	1,387 (10.7)	1,829 (10.4)
Yes	11,551 (89.3)	15,688 (89.6)
Chemotherapy		
No	1,820 (14.1)	3,143 (17.9)
Yes	11,118 (85.9)	14,374 (82.1)
Hormonal therapy		
No	5,276 (40.8)	4,532 (25.9)
Yes	7,662 (59.2)	12,985 (74.1)
Mastectomy		
Radiation		
No	7,827 (35.6)	12,322 (37.1)
Yes	14,154 (64.4)	20,857 (62.9)
Chemotherapy		
No	2,815 (12.8)	4,578 (13.8)
Yes	19,166 (87.2)	28,601 (86.2)
Hormonal therapy		
No	9,507 (43.3)	9,873 (29.8)
Yes	12,474 (56.8)	23,306 (70.2)

Percentages may not add to 100.0% due to rounding

Supplementary Table 2: Invasive tumor sizes among patients evaluated in matched invasive tumor size analysis

Characteristic	IDC alone (n = 162,065) No. (%)	IDC+DCIS (n = 260,162) No. (%)	Total (n = 422,227) No. (%)	p value
Size of invasive component:				<0.001
<1 cm	32,578 (20.1)	63,914 (24.6)	96,492 (22.9)	
1 to <2 cm	59,756 (36.9)	106,484 (40.9)	166,240 (39.4)	
2 to <3 cm	34,911 (21.5)	52,971 (20.4)	87,882 (20.8)	
3 to <4 cm	17,761 (11.0)	20,511 (7.9)	38,272 (9.1)	
4 to <5 cm	9,031 (5.6)	9,027 (3.5)	18,058 (4.3)	
5 to <7 cm	8,028 (5.0)	7,255 (2.8)	15,283 (3.6)	

Reference group: <1 cm

Abbreviations: IDC, invasive ductal carcinoma; IDC+DCIS, invasive ductal carcinoma with ductal carcinoma in situ

Percentages may not add to 100.0% due to rounding

Supplementary Table 3: Extent of DCIS among patients with negative margins

Characteristic	IDC alone (n = 168,838) No. (%)	IDC+DCIS (n = 44,563) No. (%)	Total (n = 213,401) No. (%)
Extent of DCIS:			
None	168,838 (100.0)	0 (0.0)	168,838 (79.1)
Low	0 (0.0)	35,629 (80.0)	35,629 (16.7)
Extensive	0 (0.0)	8,934 (20.1)	8,934 (4.2)

Abbreviations: ER, estrogen receptor; IDC, invasive ductal carcinoma; IDC+DCIS, invasive ductal carcinoma with ductal carcinoma in situ; PR, progesterone receptor

Percentages may not add to 100.0% due to rounding

Supplementary Table 4: Biologic subtypes among patients evaluated in subset analysis

Characteristic	IDC alone (n = 93,314) No. (%)	IDC+DCIS (n = 190,956) No. (%)	Total (n = 284,270) No. (%)	p value
Biologic subtype:				<0.001
ER+ or PR+, Her2-	60,384 (64.7)	136,449 (71.5)	196,833 (69.2)	
Triple negative	19,482 (20.9)	20,545 (10.8)	40,027 (14.1)	
Her2+	13,448 (14.4)	33,962 (17.8)	47,410 (16.7)	

Reference group: ER+ or PR+, Her2-

Abbreviations: ER, estrogen receptor; IDC, invasive ductal carcinoma; IDC+DCIS, invasive ductal carcinoma with ductal carcinoma in situ; PR, progesterone receptor

Percentages may not add to 100.0% due to rounding

Supplementary Figure 1

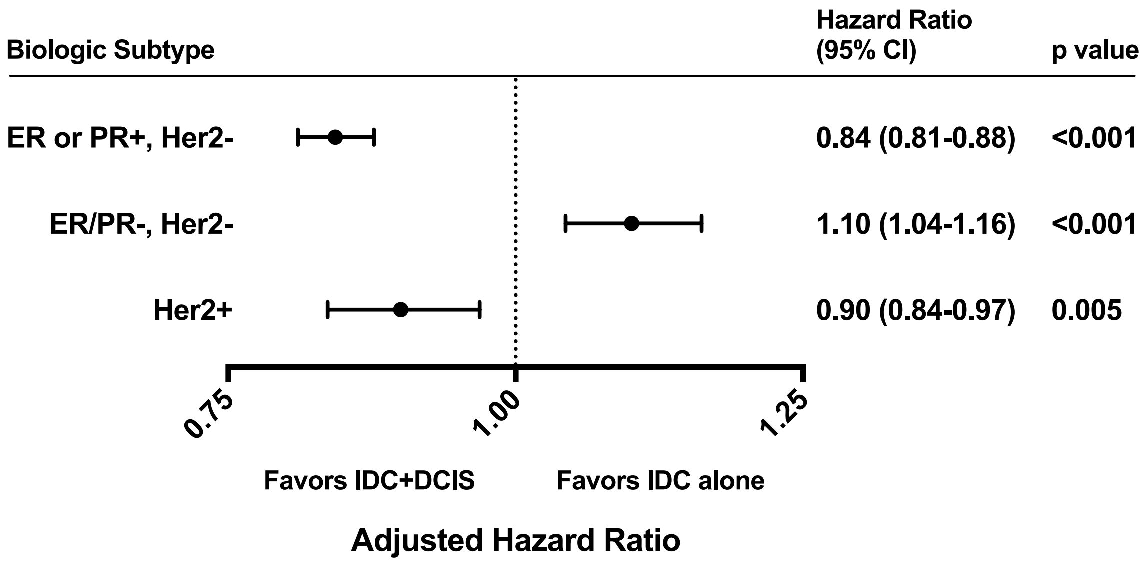

Supplement: Supplementary file 1 — Supplementary Information [file 41598_2019_46309_MOESM1_ESM.pdf]
